# Razão de mortalidade hospitalar padronizada: limites e potencialidades do indicador para a avaliação do desempenho hospitalar no Sistema Único de Saúde, Brasil 

**DOI:** 10.1590/0102-311XPT080723

**Published:** 2024-02-26

**Authors:** Marla Presa Raulino Schilling, Margareth Crisóstomo Portela, Mônica Martins

**Affiliations:** 1 Escola Nacional de Saúde Pública Sergio Arouca, Fundação Oswaldo Cruz, Rio de Janeiro, Brasil.

**Keywords:** Qualidade da Assistência à Saúde, Mortalidade Hospitalar, Avaliação de Resultados, Ajuste de Risco, Quality of Health Care, Hospital Mortality, Outcomes Assessment, Risk Adjustment, Calidad de la Atención de Salud, Mortalidad Hospitalaria, Evaluación de Resultados, Ajuste de Riesgo

## Abstract

Análises comparativas, baseadas em indicadores de desempenho clínico, para monitorar a qualidade da assistência hospitalar vêm sendo realizadas há décadas em vários países, com destaque para a razão de mortalidade hospitalar padronizada (RMHP). No Brasil, ainda são escassos os estudos e a adoção de instrumentos metodológicos que permitam análises regulares do desempenho das instituições. O objetivo deste artigo foi explorar o uso da RMHP para a comparação do desempenho dos hospitais remunerados pelo Sistema Único de Saúde (SUS). O Sistema de Informações Hospitalares foi a fonte de dados sobre as internações de adultos realizadas no Brasil entre 2017 e 2019. A abordagem metodológica para estimar a RMHP foi adaptada aos dados disponíveis e incluiu as causas de internação (diagnóstico principal) responsáveis por 80% dos óbitos. O número de óbitos esperados foi estimado por um modelo de regressão logística que incluiu variáveis preditoras amplamente descritas na literatura. A análise foi realizada em duas etapas: (i) nível da internação e (ii) nível do hospital. O modelo final de ajuste de risco apresentou estatística C de 0,774, valor considerado adequado. Foi observada grande variação da RMHP, especialmente entre os hospitais com pior desempenho (1,54 a 6,77). Houve melhor desempenho dos hospitais privados em relação aos hospitais públicos. Apesar de limites nos dados disponíveis e desafios ainda vislumbrados para a sua utilização mais refinada, a RMHP é aplicável e tem potencial para se tornar um elemento importante na avaliação do desempenho hospitalar no SUS.

## Abstract (en)

Comparative analyses based on clinical performance indicators to monitor the quality of hospital care have been carried out for decades in several countries, most notably the hospital standardized mortality ratio (HSMR). In Brazil, studies and the adoption of methodological tools that allow regular analysis of the performance of institutions are still scarce. This study aimed to assess the use of HSMR to compare the performance of hospitals funded by the Brazilian Unified National Health System (SUS). The Hospital Information System was the source of data on adult hospitalizations in Brazil from 2017 to 2019. The methodological approach to estimate HSMR was adapted to the available data and included the causes of hospitalization (main diagnosis) responsible for 80% of deaths. The number of expected deaths was estimated using a logistic regression model that included predictor variables widely described in the literature. The analysis was conducted in two stages: (i) hospitalization level and (ii) hospital level. The final risk adjustment model showed a C-statistic of 0.774, which is considered adequate. The variation in HSMR was wide, especially among the worst-performing hospitals (1.54 to 6.77). Private hospitals performed better than public hospitals. Although the limits of the available data and the challenges still face its more refined use, HSMR is applicable and has the potential to become an important tool for assessing hospital performance in the SUS.

## Resumen

Durante décadas se han realizado en varios países análisis comparativos basados en indicadores de desempeño clínico para monitorear la calidad de la atención hospitalaria, con énfasis en la razón de mortalidad hospitalaria estandarizada (RMHE). En Brasil, aún son escasos los estudios y la adopción de instrumentos metodológicos que permitan análisis regulares del desempeño de las instituciones. El objetivo fue explorar el uso de la RMHE para comparar el desempeño de los hospitales remunerados por el Sistema Único de Salud (SUS). El Sistema de Información Hospitalaria fue la fuente de datos sobre las hospitalizaciones de adultos realizadas en Brasil entre el 2017 y el 2019. El enfoque metodológico para estimar la RMHE se adaptó a los datos disponibles e incluyó las causas de hospitalización (diagnóstico principal) responsables del 80% de las muertes. El número de muertes esperadas se estimó mediante un modelo de regresión logística que incluyó variables predictoras ampliamente descritas en la literatura. El análisis se realizó en dos etapas: (i) nivel de la hospitalización y (ii) nivel del hospital. El modelo final de ajuste de riesgo presentó una estadística C de 0,774, valor considerado adecuado. Se observó una gran variación en la RMHE, especialmente entre los hospitales con peor desempeño (1,54 a 6,77). Hubo un mejor desempeño de los hospitales privados en comparación con los hospitales públicos. A pesar de las limitaciones de los datos disponibles y de los desafíos aún previstos para su uso más refinado, la RMHE es aplicable y tiene el potencial de convertirse en un elemento importante en la evaluación del desempeño hospitalario en el SUS.

## Introdução

Mensurar o desempenho dos serviços e sistemas de saúde é central nos esforços nacionais e locais para: melhorar a qualidade do cuidado; mitigar desigualdades na oferta, no acesso e nos resultados dos serviços; reduzir a variação indesejável da prática clínica; e aumentar a prestação de contas de prestadores e da rede em uma área geográfica ^1^. Avanços e intervenções, nesse sentido, são tarefas complexas e requerem continuidade. Nessa perspectiva, análises baseadas na comparação de indicadores de desempenho para monitorar a qualidade do cuidado vêm sendo aplicadas há décadas, com ênfase no cuidado hospitalar ^2^.

A mortalidade hospitalar é um critério de medição da efetividade do cuidado ^3^, uma das importantes dimensões do desempenho de serviços de saúde. Amplamente utilizada para a comparação de hospitais, requer que se leve em conta a influência das características demográficas e clínicas dos pacientes ^4^^,^^5^. É esperado, por exemplo, que pacientes mais graves tenham mais chances de irem a óbito, independentemente da qualidade do cuidado. Portanto, a aplicação de um indicador de mortalidade hospitalar depende do seu ajuste ao risco dos pacientes, que pode estar relacionado ao sexo biológico, à idade, à presença de morbidades e ao perfil de casos (*case-mix*) atendidos pelos hospitais.

Dessa forma, a taxa de mortalidade hospitalar ajustada constitui uma ferramenta de rastreamento para discriminar hospitais que potencialmente estariam prestando serviços de qualidade inferior ao padrão esperado ^3^^,^^4^. Entre as aplicações possíveis da taxa de mortalidade hospitalar ajustada, destaca-se o uso de abordagens globais, como a razão de mortalidade hospitalar padronizada (RMHP), proposta por Jarman et al. ^6^, ou abordagens específicas por condição clínica ou intervenções cirúrgicas, tratadas na literatura do ponto de vista da sua validade e utilidade para a tomada de decisão ^7^^,^^8^.

A RMHP corresponde ao quociente entre mortalidade observada e mortalidade esperada, considerando as características dos pacientes, e permite uma correção para diferenças nos *case-mix* dos hospitais ^6^. Inicialmente utilizada na Inglaterra, tem sido adaptada e aplicada em diversos países, desde o fim dos anos 1990 ^6^^,^^9^, tornando-se um indicador reconhecido como medida da qualidade hospitalar ^10^^,^^11^^,^^12^^,^^13^, embora se levantem questões acerca de sua validade ^7^^,^^14^, seja pelos limites das variáveis empregadas no ajuste de risco para capturar a gravidade dos casos ^15^, seja pela possibilidade de os resultados gerarem a seleção de pacientes por hospitais ^16^. Entre os atrativos da RMHP, estão a inclusão, na sua construção, de 80% das causas de óbito e a possibilidade de apreender a variação no desempenho dos hospitais e identificar fatores explicativos passíveis de intervenção. À luz desse debate, o uso recomendado da RMHP ancora-se nas avaliações de melhorias ao longo do tempo, sobretudo para captar o efeito de determinadas intervenções ^6^. Ou seja, é prescrito um uso mais educacional que punitivo, privilegiando-a como instrumento de monitoramento e aprendizado organizacional em detrimento de inseri-la em mecanismos de pagamento por desempenho ^17^.

No Brasil, estudos têm explorado o uso da mortalidade hospitalar como indicador do desempenho ^18^, mas iniciativas de monitoramento contínuo ainda são recentes, esbarrando, entre outros elementos, na disponibilidade de dados administrativos compatíveis com o adequado ajuste de risco. Características clínicas das internações realizadas no setor privado são raramente disponibilizadas, enquanto o Sistema Único de Saúde (SUS) passou a permitir o registro de mais de um diagnóstico secundário somente em 2015 - hoje é permitida a inserção de até nove diagnósticos secundários. Além disso, fazem-se necessários a incorporação e o aprimoramento de metodologias que propiciem mais sensibilidade na comparação de hospitais e identificação daqueles com pior desempenho.

A razão entre óbitos observados e esperados, preditos a partir de modelo de regressão logística, foi aplicada em estudo publicado em 2004, voltado para a classificação de desempenho de hospitais do SUS que realizavam revascularização do miocárdio no Brasil ^19^. Também foi aplicada, em estudo publicado em 2010, com vistas ao ranqueamento de hospitais no Rio Grande do Sul, por Gomes et al. ^20^, que analisaram internações pelo SUS nas especialidades de clínica médica e cirúrgica. O uso explícito do termo RMHP para a análise das internações brasileiras, entretanto, foi pioneiramente aplicado por Machado et al. ^21^^,^^22^ para avaliar o desempenho dos hospitais segundo o arranjo de financiamento, incluindo internações pelo SUS ou não. Esse estudo indicou que a RMHP adaptada era aplicável às informações brasileiras disponíveis e potencialmente útil para a análise e o monitoramento do desempenho hospitalar; contudo, deteve-se sobre os dados do Rio Grande do Sul e de São Paulo, devido à qualidade das informações ^22^. No nosso conhecimento, no âmbito nacional, uma única aplicação da RMHP foi realizada, recortada para hospitais selecionados, entre os quais alguns do Estado do Rio de Janeiro ^23^.

O SUS é responsável por cerca de 70-75% da assistência hospitalar no país, com predominância de unidades de pequeno porte, baixa complexidade tecnológica e baixa capacidade de resolutividade. Há grande variação na qualidade do cuidado prestado, sendo fundamental apreendê-la, identificando aspectos que necessitam de intervenção para reduzir a variação indesejada e melhorar o cuidado prestado de forma global.

Assim, este trabalho teve o objetivo de avançar na exploração do potencial de uso da RMHP para a comparação do desempenho hospitalar no Brasil, com a apropriação dos gráficos de funil, adequação metodológica e identificação de limites no contexto do SUS. Nessa perspectiva, analisa o desempenho clínico dos hospitais remunerados pelo SUS entre 2017 e 2019, levando em conta características dos pacientes e da estrutura e processos de cuidado das organizações hospitalares.

## Metodologia

### Delineamento do estudo

Estudo observacional com recorte transversal, detendo-se sobre as internações de adultos reembolsadas pelo SUS, com base em dados secundários de acesso público. A avaliação da efetividade do cuidado hospitalar baseou-se na exploração da RMHP, seguindo uma metodologia adaptada da proposta de Jarman et al. ^6^.

### Fonte de dados e universo de estudo

A fonte de dados utilizada foi o Sistema de Informações Hospitalares (SIH), base que contém informações acerca das internações hospitalares reembolsadas pelo SUS. Para a construção da base de dados, foram extraídos os registros relativos às Autorizações de Internação Hospitalar (AIH) do tipo “normal” dos anos de 2017, 2018 e 2019; os meses de janeiro a maio de 2020 foram consultados para capturar internações do ano anterior. O período foi selecionado por ser o mais recente antes da pandemia de COVID-19.

Para definir o subconjunto de internações de interesse para o estudo, aquelas responsáveis por 80% dos óbitos, partiu-se, inicialmente, da inclusão de observações relativas a pacientes entre 18 e 99 anos, e da exclusão de internações decorrentes de motivos obstétricos para as quais o óbito hospitalar é um evento sentinela, raro e, portanto, com menor validade para mensurar o desempenho hospitalar. Ademais, foram excluídos os registros cujo motivo de saída indicava encerramentos administrativos.

**Figura 1:**
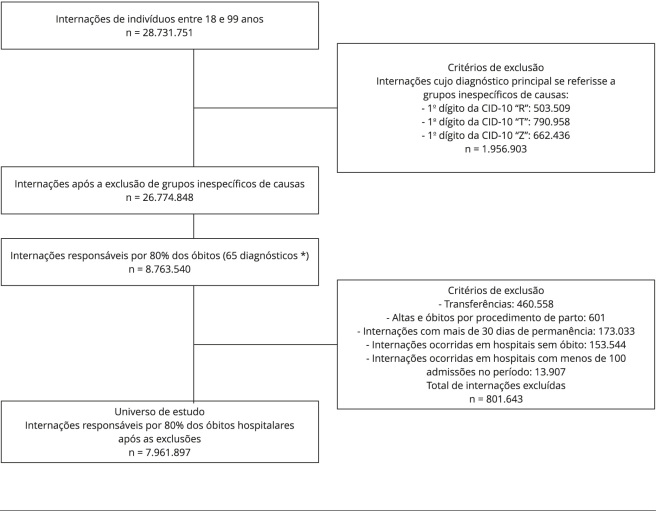
Universo do estudo - fluxograma da aplicação dos critérios de seleção para a análise da mortalidade hospitalar padronizada.

Conforme mostrado na Figura 1, considerando-se as 28.731.751 internações de indivíduos de 18 a 99 anos, selecionadas entre 2017 e 2019, foram descartadas aquelas codificadas em categorias inespecíficas da 10ª revisão da Classificação Internacional de Doenças (CID-10), incluindo as categorias diagnósticas cujo primeiro dígito era “R”, “T” ou “Z”, descritas nos capítulos XVIII, XIX e XXI. Embora a metodologia de Jarman et al. ^6^ exclua categorias diagnósticas cujo primeiro código da CID-10 inicie por “V”, “X” e “Y”, do capítulo XX, esses não foram encontrados no banco. Após a exclusão desses grupos inespecíficos de causas, o banco de dados apresentou 26.774.848 internações.

CID-10: 10ª revisão da Classificação Internacional de Doenças. * Códigos da CID-10 dos diagnósticos principais responsáveis por 80% dos óbitos hospitalares: A41, J18, I64, I50, J15, J96, I21, A49, S06, N18, J44, K92, C34, N39, C50, N17, A31, C16, I46, A48, K56, C18, I61, S72, I63, J81, C61, K70, C15, C25, K74, E14, E46, C53, I20, C22, I60, J12, G91, J90, G45, A40, E10, C71, D64, I74, C20, C80, B20, J93, K76, I26, K72, K65, L89, K63, A46, S27, C56, I71, I44, C32, S36, K80, E86.

Em seguida, foram selecionadas as internações cujas causas (diagnósticos principais), definidas em termos dos três primeiros dígitos da CID-10 e ordenadas de modo decrescente segundo a ocorrência de óbito, corresponderam a 80% das mortes hospitalares. Desse estágio, resultaram 8.763.540 internações, referentes a 65 categorias diagnósticas (Figura 1).

Além da delimitação da faixa etária (18 a 99 anos), não estabelecida por Jarman et al. ^6^, na última etapa de conformação do universo de estudo, foram excluídas as internações cujo motivo de saída indicou: transferências (códigos da variável “cobrança” 31 e 32, no SIH): casos residuais de altas e óbitos relacionados ao parto (código “cobrança” 61, 62, 63, 64, 65, 66 e 67); e internações cujo tempo de permanência foi superior a 30 dias, outra adaptação feita à metodologia de Jarman et al. ^6^. Para fins de análise, foram excluídos os hospitais que registraram menos de 100 admissões, e internações ocorridas em hospitais que não registraram óbito no período do estudo (Figura 1). O universo do estudo resultou em 7.961.897 internações, correspondendo a, aproximadamente, 28% das internações de indivíduos adultos no período.

### Análise dos dados

A RMHP compara o número de óbitos observados com o número que seria esperado, estimado por meio de um modelo de ajuste de risco no nível de cada internação. Para compor o denominador da RMHP, definido pelo número de óbitos esperados, utilizou-se a técnica de regressão logística, com uma estratégia de adição sequencial das variáveis independentes para a predição de ocorrência de óbito, com vistas a apreender a contribuição de cada adição na estatística C. O modelo de base foi composto por sexo e idade, sendo as demais variáveis disponíveis, com relevância na predição do risco de óbito atestada na literatura ^5^, incluídas uma a uma. No modelo final, o número esperado de óbitos foi estimado com base nas seguintes variáveis: idade (estratificada em 18-49, 50-59, 60-69, 70-79, 80-89 e 90-99 anos); sexo; diagnóstico principal (65 categorias); presença de comorbidade (diagnóstico secundário 1 a 9 do SIH), mensurada por meio do índice de Charlson (IC) ^24^^,^^25^; e caráter da internação, classificada em eletiva ou de emergência. Todas essas variáveis são reconhecidas como fatores impactantes sobre o prognóstico do caso ^5^. A variável caráter da internação foi dicotomizada de forma que as urgências englobam também os acidentes no local de trabalho ou a serviço da empresa, acidentes no trajeto para o trabalho, outros tipos de acidente de trânsito, outros tipos de lesões e envenenamentos por agentes químicos ou físicos. Os 65 diagnósticos principais responsáveis por 80% dos óbitos foram categorizados segundo a mortalidade bruta de cada um, sendo considerado como categoria de referência o código K80 (colelitíase) da CID-10, que apresentou uma mortalidade bruta de 0,6%.

No cálculo do IC, o algoritmo proposto por Quan et al. ^24^ foi utilizado. O IC foi calculado a partir dos nove diagnósticos secundários disponíveis no SIH, considerando somente aqueles que eram preexistentes à internação (tipo de diagnóstico secundário = 1) e categorizados em 0, 1 e ≥ 2. O índice de Elixhauser ^25^ também foi testado, porém não foi mantido no modelo final por não ter apresentado significância estatística.

Na adaptação da proposta de Jarman et al. ^6^^,^^9^, considerou-se a disponibilidade de informação e os estudos nacionais precedentes ^22^^,^^23^. Entretanto, variáveis sobre o estado socioeconômico, tempo de permanência, ano e mês de admissão, admissão para cuidado paliativo (CID-10: Z51.5) e fonte de admissão (casa, outro hospital etc.) não foram incluídas. Vale destacar que, no caso do diagnóstico principal, optou-se, por razão pragmática e de compreensibilidade, pelo uso da CID-10. Apesar de o SIH dispor sobre a “raça/cor” do paciente na internação, uma variável *proxy* da situação socioeconômica, escolheu-se analisá-la somente como variável explicativa no âmbito da internação e não como uma variável de ajuste, o que poderia criar a impressão de que é aceitável uma mortalidade mais alta para as populações mais carentes. Como o tempo de permanência pode refletir gravidade ou complicação decorrente de problemas do cuidado ou ainda disponibilidade de serviços de longa permanência ou reabilitação, preferiu-se não o incluir, considerando problemas na qualidade e/ou suficiência da informação sobre comorbidades nos dados brasileiros. Decidiu-se, ainda, não incluir mês e ano de admissão no modelo de ajuste de risco por não ter sido observada variação significativa na mortalidade hospitalar ao longo dos três anos avaliados. Quanto ao ajuste para internações em cuidados paliativos, é possível que haja variação considerável entre os hospitais na codificação desses cuidados e ainda uma baixa completude para essa informação, o que justifica a não inclusão dessa variável no modelo de ajuste de risco. Já a fonte de admissão não pôde ser incluída por não constar no banco de dados do SIH. Também vale destacar que se optou por testar modelos incluindo todas as categorias diagnósticas selecionadas (65 no total), diferentemente das adaptações mais recentes ^9^, que estimam o número de óbitos preditos em modelos específicos, para cada agrupamento de categoria diagnóstica, empregando as mesmas variáveis de risco, e depois contabilizando os óbitos preditos.

A capacidade discriminativa do modelo final foi testada com base na estatística C, área sob a curva ROC (*receiver operating characteristics*). Os valores do teste assumem uma faixa que varia de 0,5 a 1,0, tendo capacidade preditiva razoável de valores iguais ou maiores que 0,7 ^26^. As análises foram realizadas utilizando o pacote estatístico SPSS 24.0 (https://www.ibm.com/).

### Avaliação da mortalidade hospitalar

A partir do modelo de ajuste de risco final estimado no nível de internação, foi criado um banco de dados agregado por estabelecimento de saúde, no qual foram computados o total de mortes ocorridas e o total de mortes preditas, além do volume de internações. A RMHP (óbitos observados/óbitos preditos) foi calculada e, para avaliar seu padrão, foram utilizados gráficos de funil. Essa razão foi, então, plotada no eixo vertical e o denominador (somatório das mortes previstas pelo ajuste de risco) foi plotado no eixo horizontal. A linha central pontilhada, representa o eixo em que o somatório das mortes observadas é igual ao somatório das mortes preditas pelo modelo (RMHP: mortes observadas/mortes esperadas = 1). Em ambos os lados dessa linha, estão plotados dois conjuntos de linhas que representam a distância de três e dois desvios padrão (DP). Os círculos azuis plotados no gráfico representam cada hospital e podem ser identificados pelo código do Cadastro Nacional de Estabelecimento de Saúde (CNES).

Um fator de sobredispersão, comumente utilizado na análise de dados de populações heterogêneas, foi aplicado aos limites do gráfico de funil com o objetivo de reduzir os efeitos de possíveis valores discrepantes, para os quais o modelo de ajuste de risco não corrige suficientemente ^27^, conforme aplicado aos dados administrativos dos Serviços Nacionais de Saúde (NHS) inglês ^28^ e escocês ^29^ e sugerido por especialistas da área ^30^. Para garantir que as estimativas da sobredispersão sejam robustas, a influência dos casos periféricos, os quais devem ser detectados pelo sistema, precisa ser minimizada por meio da *winsorização*, que consiste em redimensionar os valores mais extremos para determinado percentil ^27^^,^^31^, que neste estudo foi de 10% em cada extremidade da distribuição.

Assim, uma implementação do gráfico de funil para razões padronizadas indiretamente e ajustadas pela sobredispersão com limites de três DP, conforme descrito por Spiegelhalter ^27^^,^^31^, classificou os hospitais em três categorias: RMHP acima do esperado, representando os hospitais com pior desempenho; RMHP compatível com o esperado, para aqueles com o desempenho aguardado; e RMHP abaixo do esperado, os hospitais com melhor desempenho. A medida de três DP tem sido tomada como o limite em gráficos de funil ^30^. Os gráficos foram contruídos no programa estatístico R (http://www.r-project.org), por meio do pacote *FunnelPlotR*^32^.

Foram calculadas as estimativas do desempenho hospitalar de acordo com os estratos de RMHP, incluindo: número de internações, mortes observadas, mortes esperadas, mortalidade bruta e mortalidade esperada. Um painel com características específicas da rede hospitalar, segundo o padrão da RMHP, considerou: grande região geográfica do país onde o hospital se localiza (Norte/Nordeste/Centro-oeste/Sudeste/Sul); natureza jurídica (público/privado/filantrópico); tempo de permanência em dias (média/DP); valor total da internação (média); e valor do uso de unidade de terapia intensiva (UTI) (média). Internações cujo tempo de permanência foi zero, foram recodificadas como um dia. Vale sublinhar que, dos 3.449 hospitais incluídos na análise, 81 apresentaram alteração no registro da natureza jurídica ao longo do período de estudo e, portanto, não foram incluídos na distribuição segundo a RMHP. No nível da internação, foram apresentadas, segundo a RMHP, as seguintes variáveis: sexo (masculino/feminino); idade (média); faixa etária em anos (18-49/50-59/60-69/70-79/80-89/90-99); raça/cor (branco/preto/pardo/amarelo/indígena); escore do IC (0/1/≥ 2); utilização de UTI (não/sim); e caráter da internação (eletiva/urgência).

Para finalizar, sublinha-se que este estudo, baseado exclusivamente em dados secundários, anônimos, de acesso público, enquadra-se em critério de dispensa de submissão e aprovação em Comitê de Ética em Pesquisa.

## Resultados

No universo das 7.961.897 internações selecionadas a partir da aplicação dos critérios de inclusão e exclusão, a média de idade dos indivíduos foi de 60,7 anos. Ao todo, 13,1% das internações resultaram em óbito. Os diagnósticos principais mais frequentes foram: pneumonia por microrganismo não especificado (CID-10 J18: 8,7%), colelitíase (CID-10 K80: 6,9%) e insuficiência cardíaca (CID-10 I50: 6,7%). Apenas 13,4% das internações tinham registro de comorbidade preexistente (variável diagnóstico secundário 1) e aproximadamente 97% não pontuaram no IC. Cerca de 50% das internações ocorreram em hospitais filantrópicos (Tabela 1).

**Tabela 1:** Características das internações hospitalares de adultos * responsáveis por 80% dos óbitos registrados (n = 7.961.897 **) no Sistema de Informações Hospitalares do Sistema Único de Saúde. Brasil, 2017-2019.

Características	n	%
Idade (anos) [média (DP)]	60,7 (18,2)		
Sexo			
Mulher	3.970.326	49,9
Homem	3.991.571	50,1
Uso de UTI		
Sim	1.036.401	13,0
Não	6.925.496	87,0
Diagnóstico principal			
J18 - Pneumonia por microrganismo não especificado	689.450	8,7
K80 - Colelitíase	550.239	6,9
I50 - Insuficiência cardíaca	534.441	6,7
N39 - Outros transtornos do trato urinário	422.864	5,3
I64 - Acidente vascular cerebral, não especificado	408.956	5,1
I20 - Angina pectoris	335.802	4,2
I21 - Infarto agudo do miocárdio	289.412	3,6
J15 - Pneumonia bacteriana não classificada em outra parte	270.055	3,4
A41 - Outras septicemias	264.973	3,3
S72 - Fratura do fêmur	254.475	3,2
Outros	3.941.230	49,5
Diagnóstico secundário 1 ***		
Sim	1.065.038	13,4
Não	6.896.859	86,6
Diagnóstico secundário 2 ***		
Sim	235.626	3,0
Não	7.726.271	97,0
Diagnóstico secundário 3 ***		
Sim	100.861	1,3
Não	7.861.036	98,7
IC			
0	7.692.917	96,6
1	140.759	1,8
≥ 2	128.221	1,6
Resultado do cuidado			
Saída	6.916.536	86,9
Óbito	1.045.361	13,1
Caráter da internação			
Eletiva	1.211.604	15,2
Emergência	6.750.293	84,8
Natureza jurídica do hospital ^#^			
Pública	3.554.578	44,6
Privada	555.215	7,0
Filantrópica	3.851.895	48,4
Tempo de permanência (dias) [média (DP); mediana] ^#^	6,0 (5,7); 4,0		

DP: desvio padrão; IC: índice de Charlson; UTI: unidade de terapia intensiva.

Fonte: Departamento de Informática do SUS ^44^.

* 18-99 anos;

** Após a exclusão de 801.643 internações (transferências = 460.558; altas e óbitos por procedimento de parto = 601; internações com mais de 30 dias de permanência = 173.033; internações ocorridas em hospitais sem registro de óbito = 153.544; internações ocorridas em hospitais com menos de 100 admissões no período do estudo = 13.907);

*** Comorbidades preexistentes;

^#^ Calculado no nível da internação.

O modelo de ajuste de risco de base, composto por sexo e idade, apresentou capacidade discriminativa de 0,648 (intervalo de 95% de confiança - IC95%: 0,647-0,649) e, com a inclusão do diagnóstico principal ao modelo, a estatística C passou para 0,766 (IC95%: 0,765-0,766). Já a introdução do IC pouco acrescentou à capacidade discriminativa do modelo (estatística C: 0,768; IC95%: 0,768-0,768). O modelo final de ajuste de risco, composto pelas variáveis sexo, idade, diagnóstico principal, IC e caráter da internação, apresentou estatística C de 0,774 (IC95%: 0,773-0,774), considerada adequada.

**Figura 2:**
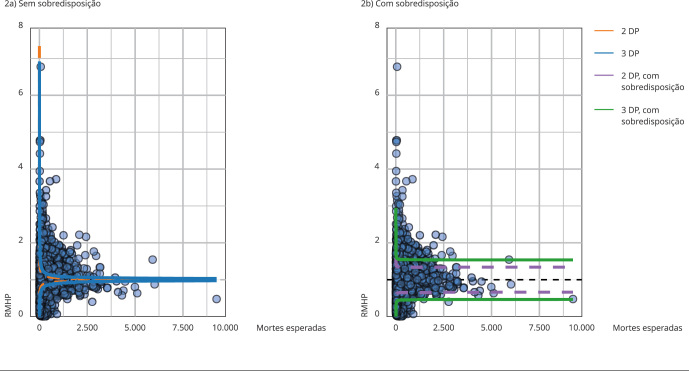
Gráfico de funil da razão de mortalidade hospitalar padronizada (RMHP).

Dos 3.449 hospitais selecionados, 2.389 apresentaram valores desviantes (*outliers*) para RMHP (Figura 2a). Ao serem plotados com sobredispersão, foram identificados 1.245 hospitais com desempenho fora do padrão esperado, ou seja, com RMHP refletindo uma mortalidade observada estatisticamente superior ou inferior à mortalidade esperada (Figura 2b). Ao todo, foram identificados 249 hospitais com pior desempenho (RMHP elevada, indicando mortalidade superior à esperada), que registraram 919.406 internações, com 216.343 mortes, sendo a mortalidade bruta de 25,6%, enquanto a esperada era de 12,9%. A RMHP nesses hospitais variou de 1,54 a 6,77. Já os 996 hospitais com melhor desempenho (RMHP baixa, indicando mortalidade inferior à esperada) registraram 855.520 internações, com 24.295 mortes, mortalidade bruta de 2,7% e esperada de 11,8%. Nos hospitais com melhor desempenho clínico, a RMHP variou de 0,01 a 0,45 (Tabela 2).

DP: desvio padrão. Fonte: Departamento de Informática do SUS ^44^. Nota: a RMHP é apresentada no eixo vertical e o número de óbitos esperados, empregado no cálculo do indicador, é apresentado no eixo horizontal. Os hospitais foram plotados sem sobredispersão (2a) e com sobredispersão (2b).

**Tabela 2:** Estimativas do desempenho dos hospitais, segundo a classificação da razão de mortalidade hospitalar padronizada (RHMP).

Variáveis	Total	RMHP		
		Abaixo do esperado * (melhor desempenho)	Compatível ao esperado * (desempenho esperado)	Acima do esperado * (pior desempenho)
Hospitais	3.449	996	2.204	249
Internações				
n	7.961.897	855.520	6.186.971	919.406
Variação	101-86.181	102-18.696	101-86.181	106-39.379
Média	2.308,46	858,96	2.807,16	3.692,39
Mortes observadas				
n	1.045.361	24.295	804.723	216.343
Variação	1-9.130	1-1.109	1-5.253	26-9.130
Média	303,09	24,39	365,12	868,85
Mortes esperadas				
n	1.045.361	99.972,21	826.561,35	118.827,44
Variação	0,68-9.262,96	6,07-2.761,63	0,68-9.262,96	9,55-5.919,20
Média	303,09	100,37	375,03	477,22
Mortalidade bruta				
%	9,95	2,69	11,47	25,59
Variação	0,04-100	0,04-8,35	0,52-64,98	4,01-100
DP	8,26	1,77	5,34	14,35
Mortalidade esperada				
%	12,62	11,82	12,95	12,92
Variação	0,36-46,01	0,68-39,60	0,36-46,01	1,60-39,17
DP	3,82	3,79	3,61	5,05
RMHP **				
%	0,77	0,22	0,88	1,99
Variação	0,01-6,77	0,01-0,45	0,17-1,71	1,54-6,77
DP	0,54	0,12	0,30	0,64

DP: desvio padrão.

Fonte: Departamento de Informática do SUS ^44^.

* Ajustado por sobredispersão com limite de três DP;

** Razão entre óbitos observados e óbitos estimados, a partir do modelo de ajuste de risco.

A Região Sul foi a que apresentou menor percentual de hospitais com desempenho ruim; dos 678 hospitais do Sul, apenas 14 (2,1%) apresentaram RMHP acima do esperado. Dos 234 hospitais privados avaliados, 126 (53,8%) apresentaram resultados melhores do que o esperado, 95 (40,6%) apresentaram desempenho compatível com o esperado, e apenas 13 (5,6%) tiveram desempenho pior do que esperado. Entre os hospitais que dispunham de UTI, para aqueles com melhor desempenho, a média dos valores pagos em média por uso de UTI foi de R$ 452,15, enquanto, entre aqueles com desempenho abaixo do esperado, a média dos valores pagos em média por uso de UTI foi de R$ 759,41 (Tabela 3).

**Tabela 3:** Características dos hospitais (n = 3.449) e das internações (n = 7.961.897), segundo o nível do desempenho com base na razão de mortalidade hospitalar padronizada (RMHP).

Nível de análise/Variáveis	Total	RMHP		
		Abaixo do esperado * (melhor desempenho)	Compatível ao esperado * (desempenho esperado)	Acima do esperado * (pior desempenho)
	n	n (%)	n (%)	n (%)
Hospitais	3.449	996 (28,9)	2.204 (63,9)	249 (7,2)
Região do país				
Norte	281	121 (43,1)	134 (47,7)	26 (9,3)
Nordeste	1.008	355 (35,2)	566 (56,2)	87 (8,6)
Centro-oeste	346	139 (40,2)	188 (54,3)	19 (5,5)
Sudeste	1.136	178 (15,7)	855 (75,3)	103 (9,1)
Sul	678	203 (29,9)	461 (68,0)	14 (2,1)
Natureza jurídica **				
Público	1.738	531 (30,6)	1.037 (59,7)	170 (9,8)
Privado	234	126 (53,8)	95 (40,6)	13 (5,6)
Filantrópico	1.396	321 (23,0)	1.015 (72,7)	60 (4,3)
Tempo de permanência [média (DP)]	5,15 (2,03)	3,92 (1,59)	5,45 (1,88)	7,39 (1,99)
Valores do reembolso [média]				
Internação	1.109,27	825,88	1.184,34	1.578,41
Uso de UTI ***	175,21	17,25	207,81	518,47
Uso de UTI ^#^	614,74	452,15	590,98	759,41
Internações	7.961.897	855.520 (10,7)	6.186.971 (77,7)	919.406 (11,5)
Sexo				
Masculino	3.991.571	398.104 (10,0)	3.115.796 (78,1)	477.671 (12,0)
Feminino	3.970.326	457.416 (11,5)	3.071.175 (77,4)	441.735 (11,1)
Idade [média]	60,7	60,0	61,0	60,0
Faixa etária (anos)				
18-49	2.069.189	261.159 (12,6)	1.542.296 (74,5)	265.734 (12,8)
50-59	1.366.085	124.864 (9,1)	1.086.428 (79,5)	154.793 (11,3)
60-69	1.714.954	154.864 (9,0)	1.371.458 (80,0)	188.632 (11,0)
70-79	1.545.643	165.002 (10,7)	1.211.779 (78,4)	188.862 (10,9)
80-89	1.011.195	118.147 (11,7)	780.918 (77,2)	112.130 (11,1)
90-99	254.831	31.484 (12,4)	194.092 (76,2)	29.255 (11,5)
Raça/Cor				
Branco	3.157.051	256.780 (8,1)	2.725.180 (86,3)	175.091 (5,5)
Preto	338.063	21.882 (6,5)	262.402 (77,6)	53.779 (15,9)
Pardo	2.624.815	340.179 (13,0)	1.895.664 (72,2)	388.972 (14,8)
Amarelo	169.444	29.728 (17,5)	121.137 (71,5)	18.579 (11,0)
Indígena	9.706	3.739 (38,5)	4.963 (51,1)	1.004 (10,3)
IC				
0	7.692.917	850.293 (11,1)	5.937.236 (77,2)	905.388 (11,8)
1	140.759	3.326 (2,4)	129.369 (91,9)	8.064 (5,7)
≥ 2	128.221	1.901 (1,5)	120.366 (93,9)	5.954 (4,6)
Utilização de UTI				
Não	6.925.496	836.051 (12,1)	5.293.088 (76,4)	796.357 (11,5)
Sim	1.036.401	19.469 (1,9)	893.883 (86,2)	123.049 (11,9)
Caráter da internação				
Eletiva	1.211.604	54.655 (4,5)	942.985 (77,8)	213.964 (17,7)
Urgência	6.750.293	800.865 (11,9)	5.243.986 (77,7)	705.442 (10,5)

DP: desvio padrão; IC: índice de Charlson; UTI: unidade de terapia intensiva.

Fonte: Departamento de Informática do SUS ^44^.

* Ajustado por sobredispersão com limite de três DP;

** Não foram incluídos os hospitais que sofreram alteração da natureza jurídica no período do estudo;

*** Para o cálculo da média, foram incluídos todos os hospitais, inclusive os que não utilizaram UTI;

^#^ Cálculo efetuado apenas para os hospitais que utilizaram UTI.

No que concerne à raça/cor, observa-se que 15,9% das internações de pretos foram realizadas em hospitais com RMHP elevada (desempenho ruim), sendo que, para brancos, esse percentual foi de 5,5%. Das internações que apresentaram maior escore no IC (≥ 2), 4,6% foram em hospitais com RMHP elevado e 1,5% em hospitais com RMHP abaixo do esperado (Tabela 3).

## Discussão

Este estudo teve caráter exploratório, valendo sublinhar o seu foco mais direcionado à apropriação e adequação de técnicas empregadas para a operacionalização do uso da RMHP do que à classificação do desempenho dos hospitais *per si*, sob olhar crítico acerca dos limites impostos pelos dados disponibilizados pelo SUS. Nesse sentido, cabe destacar a necessidade vivenciada de se fazer escolhas metodológicas plausíveis no contexto do SUS, a utilização dos gráficos de funil e o reconhecimento dos limites impostos pela qualidade dos dados hospitalares disponibilizados.

O modelo final de ajuste de risco desenvolvido para a construção da RMHP, adaptado a partir da metodologia de Jarman et al. ^6^, apresentou razoável capacidade discriminativa (C = 0,774). Apesar das adaptações nos critérios de seleção, que foram necessárias aos dados administrativos brasileiros, a inclusão das 65 categorias diagnósticas responsáveis por 80% dos óbitos hospitalares é semelhante à encontrada em outros países, como Austrália (72 categorias) e Canadá (74 categorias) ^33^^,^^34^. Neste estudo, optou-se por trabalhar com as categorias diagnósticas a partir dos códigos CID-10, disponibilizados no SIH; embora, em outros países - como a Inglaterra ^28^, que utiliza o indicador sumário de mortalidade hospitalar (*summary hospital mortality indicator* - SHMI), e os Países Baixos ^9^^,^^35^ -, tais códigos se convertam em categorias do Sistema de Classificação Clínica (*Clinical Classification System* - CCS), o que inviabiliza a comparação direta de resultados. O CCS engloba 260 categorias diagnósticas mutuamente exclusivas e atribui cada código da CID-10 a uma dessas categorias, auxiliando a produção de relatórios de mortalidade ^36^. Contudo, como o CCS é pouco conhecido no Brasil, sua utilização poderia comprometer a compreensibilidade dessa estratégia metodológica pelos distintos atores no contexto brasileiro.

Quanto às variáveis incluídas no modelo final de ajuste de risco, destaca-se a similaridade com a opção inglesa, o SHMI, que é ajustado por sexo, idade, IC e tipo de admissão (na terminologia do SIH, caráter da internação), além das variáveis ano, mês da internação e peso ao nascer, para grupos de diagnóstico perinatal ^28^. Segundo a Escola de Saúde e Pesquisas Relacionadas da Universidade de Sheffield (Inglaterra), ao usar apenas idade, sexo, IC e tipo de admissão, é possível fornecer um modelo simples e estável ^37^. O uso de um maior número de variáveis de ajuste de risco pode melhorar o poder preditivo dos modelos, mas não acrescenta discriminação adicional no desempenho dos hospitais ^38^.

Na Austrália, além das variáveis de ajuste de risco utilizadas neste estudo, é incluído o *status* de transferência e o tempo de permanência ^33^. Entretanto, a inclusão do tempo de permanência nos modelos de risco tem sido amplamente discutida, devido ao efeito ambíguo que pode exercer sobre a mortalidade. Além dos motivos clínicos que levam a um maior ou menor tempo de internação, o estímulo a práticas gerenciais mais eficientes pode reduzir esse período e, inclusive, estimular altas precoces, não refletindo, nesse caso, em gravidade ou melhoria na qualidade do cuidado. A disponibilidade de leitos de longa permanência e unidades de reabilitação também pode reduzir ou prolongar o tempo de permanência ^39^^,^^40^. Outra questão a se considerar são as mortes ocorridas no primeiro dia de internação, que podem representar a gravidade do caso, barreiras de acesso ou inadequação assistencial da unidade de emergência ^22^. Assim, optou-se por não incluir a variável tempo de permanência no modelo de ajuste de risco, além de terem sido excluídas as internações com mais de 30 dias, na tentativa de privilegiar a relação entre processo de cuidado e resultado (óbito), conforme ponderado por Machado et al. ^22^.

Nos Países Baixos, o Instituto Central de Estatísticas (CBS) recomenda o ajuste por “situação socioeconômica” ^35^, conforme já tinha sido feito por Jarman et al. ^9^ em estudo publicado em 2010, em que consideraram a variável privação social (*social deprivation*) no ajuste de risco. Já a atual metodologia para a construção do SHMI, na Inglaterra, não faz nenhum ajuste para privação social. Indicadores contextuais sobre a porcentagem de internações e mortes por nível de privação (do mais carente ao menos) são produzidos apenas para apoiar a interpretação do SHMI ^28^.

Outra questão a ser discutida é a inclusão ou não dos hospitais especializados. Para a construção do SHMI, os ingleses incluem apenas os hospitais não especializados ^28^, assim como os canadenses para o cálculo da RMHP ^34^. Já os holandeses incluem hospitais especializados, desde que tenham realizado mais de 30% de internações agudas (curta permanência) ^35^, e os australianos analisam instituições de cuidados agudos e cuidados geriátricos de avaliação, gerenciamento e manutenção ^33^.

Neste estudo não foram excluídos os hospitais especializados, dado que, no Brasil, importantes instituições especializadas, vinculadas ao SUS, como o Instituto do Coração, são responsáveis por grande parte das internações associadas aos motivos de internação incluídos no cálculo da RMHP (p.ex.: I50/insuficiência cardíaca; I44/bloqueio atrioventricular e do ramo esquerdo; I46/parada cardíaca). Novos estudos avaliando o impacto da exclusão dos hospitais especializados no cálculo da RMHP devem ser conduzidos.

Assim, modelos de ajuste de risco que buscam minimizar os efeitos dos fatores de confusão, que, independentemente do processo de cuidado, interferem no resultado, têm sido propostos por diversas agências ao redor do mundo ^28^^,^^33^^,^^34^. É unânime a indicação de que a metodologia utilizada para gerar o modelo deve ser revisada periodicamente para garantir robustez e comparações adequadas para cada ponto no tempo ^41^.

No que concerne aos resultados da análise realizada, foi observada uma grande variação da RMHP, especialmente entre os hospitais com pior desempenho (RMHP entre 1,54 e 6,77), nos quais foram realizadas 11,5% das internações. Esse achado é compatível com o observado em estudo que analisou a variação do desempenho hospitalar em São Paulo e no Rio Grande do Sul. Machado et al. ^22^ utilizaram, para ordenar a RMHP, o corte de percentis (percentil 20; percentil de 30 a 70; percentil 80) e apontaram grande variação da RMHP, de 1,2 a 2,4, e alto volume de internações para aqueles com pior desempenho (percentil 80), ilustrando a importância de uma análise continuada e aprofundada do indicador.

Os resultados apontaram, ainda, um melhor desempenho dos hospitais privados em relação aos hospitais públicos, como no estudo de Machado et al. ^21^; contudo, o papel dos hospitais privados foi circunscrito no universo de estudo (7% das internações) comparativamente aos hospitais públicos e filantrópicos. Vale destacar que, nesse universo, estão os hospitais públicos federais de ensino por serem designados como entidades empresariais, por conta da gestão por empresas públicas - Hospital Nossa Senhora da Conceição e Empresa Brasileira de Serviços Hospitalares (EBSERH). Essas unidades desenvolvem, além do cuidado, atividades de ensino e pesquisa para as quais contam com insumos e recursos mais complexos, necessários ao perfil de casos tratados e à essas atividades. A reclassificação desses hospitais poderia ser considerada em outros estudos, embora não se avalie que pudessem modificar substantivamente os achados apresentados. Ademais, os achados relativos às médias de reembolso do SUS dão indícios de que os piores desempenhos ocorrem nos hospitais onde se realizam cuidados mais complexos, o que pode sugerir limites do ajuste de risco.

Os resultados encontrados podem refletir uma série de fatores, como características dos pacientes atendidos (problemas no modelo de ajuste de risco), erros e subnotificação nas bases de dados, variação ao acaso, ou a qualidade do processo de cuidado. Vale sublinhar que uma única medida aparentemente alta da RMHP não é evidência suficiente para concluir que o serviço desempenha aquém do esperado ^29^. Portanto, reitera-se a necessidade de aprimorar o desenvolvimento de ferramentas que permitam monitorar o desempenho dos serviços de saúde e, consequentemente, estimulem a melhoria da qualidade do cuidado, reduzindo desigualdades entre as diferentes regiões do país e entre os hospitais públicos, privados e filantrópicos, melhorando a efetividade do SUS.

### Limites

Considerando a natureza multidimensional dos conceitos de qualidade e desempenho, e a impossibilidade de se encontrar uma medida capaz de mensurar todas as dimensões, o próprio uso da RMHP pode ser considerado uma limitação do estudo, devendo ser compreendida como uma aproximação indireta da qualidade, preferencialmente voltada para delinear ações de melhoria ^41^^,^^42^. Além disso, há outros limites, como: a inclusão apenas dos óbitos intra-hospitalares, pois, a depender da política de admissão e alta, podem ocorrer após a alta, embora seja necessário definir o intervalo de tempo adequado ^40^; as questões associadas à robustez do ajuste de risco e aos métodos estatísticos empregados; as ferramentas para testar a capacidade preditiva do modelo; a interpretação da variação entre os hospitais; e os métodos gráficos de apresentação das informações.

Quanto aos óbitos intra-hospitalares, a abordagem de Jarman et al. ^6^ não computou aqueles ocorridos até 30 dias após a alta. Embora existam fortes correlações entre os modelos intra-hospitalar e o que abrange os óbitos ocorridos até 30 dias após a alta ^43^, o impacto sobre hospitais com valores desviantes (*outliers*) pode ser significativo ^41^. Atualmente, na Inglaterra, o SHMI inclui tanto a morte intra-hospitalar como a morte ocorrida até 30 dias após a alta ^28^. Apesar da possibilidade de os impactos nos cuidados hospitalares se tornarem aparentes após a alta, a decisão sobre a definição de mortalidade em relação ao tempo é determinada por questões pragmáticas, como a viabilidade de acesso a dados vinculados de óbito da população ^30^^,^^41^, além da dificuldade operacional de relacionar bancos de dados sem um identificador único, como é o caso dos sistemas de saúde brasileiro de acesso público.

Outra limitação importante com relação ao modelo de ajuste de risco diz respeito à inacessibilidade a informações sobre a gravidade da doença principal, que em geral não estão disponíveis nos dados administrativos. Alguns autores ^7^^,^^8^^,^^13^^,^^14^ destacam que a inclusão de diversas e diferentes causas agrega grande heterogeneidade quanto ao risco e prognóstico, podendo causar problemas quanto à validade atribuível ^42^. Além disso, apesar da inclusão de nove diagnósticos principais no SIH desde 2015, esses campos apresentaram completude baixa, o que pode ter impactado os escores do IC, a precisão do ajuste de risco, e, consequentemente, a estimativa dos óbitos preditos. A variável comorbidade vem sendo apontada como a mais importante em modelos de ajuste de risco, após idade e tipo de admissão ^43^, o que reforça a necessidade de adoção de medidas que estimulem melhorias no preenchimento das informações referentes, principalmente, às comorbidades.

Ainda, merecem atenção ponderações sobre o que assumir como referência na modelagem de ajuste de risco e na consequente estimativa de óbitos esperados. Este trabalho optou pela inclusão de um conjunto abrangente de hospitais, em que a estimativa de óbitos representa possivelmente um padrão médio geral. Entretanto, critérios mais restritivos no sentido da adoção de padrões estabelecidos a partir de hospitais selecionados, reconhecidos por melhores resultados do que a média geral, podem ser incorporados.

## Conclusão

Em suma, há problemas acerca do uso da RMHP ^6^, mas também é preciso reconhecer a sua utilidade em análises de melhorias na qualidade do cuidado. Nessa perspectiva, apesar das limitações das informações sobre produção hospitalar no Brasil, o modelo de risco aplicado à RMHP no âmbito nacional oferece uma visão ampla do padrão e variabilidade do desempenho da rede hospitalar do SUS. Contudo, melhorias na qualidade da informação concernente à cobertura do universo de internações brasileiras, ao espectro das informações e à precisão do registro de dados são urgentes, potencializando análises agregadas segundo âmbitos geográficos, esferas de gestão e grupos socialmente mais vulneráveis no tocante à variação na efetividade do cuidado hospitalar recebido. Embora conhecida, a carência de informação no contexto recente da pandemia deixou evidente a seriedade e as consequências dessas lacunas.
